# Sustainability Planning for a Community Network to Increase Participation in Evidence-Based Lifestyle Change Programs: A Mixed-Methods Approach

**DOI:** 10.3390/ijerph21040463

**Published:** 2024-04-10

**Authors:** Maura M. Kepper, Katherine A. Stamatakis, Ariel Deitch, Ally Terhaar, Emerald Gates, Gabrielle Cole, Carolyn S. French, Amy Hampton, Lauren Anderson, Amy A. Eyler

**Affiliations:** 1 Prevention Research Center, Brown School, Washington University in St. Louis, St. Louis, MO 63130, USAaeyler@wustl.edu (A.A.E.); 2 Department of Epidemiology and Biostatistics, College for Public Health and Social Justice, Saint Louis University, St. Louis, MO 63104, USA; 3 St. Louis County Department of Public Health, St. Louis, MO 63134, USA; egates@stlouiscountymo.gov; 4 Fit and Food Connection, St. Louis, MO 63136, USA; 5 Gateway Region YMCA, St. Louis, MO 63103, USA; 6 Missouri Department of Health and Senior Services, Bureau of Cancer and Chronic Disease Prevention, Jefferson City, MO 65109, USA; amy.hampton@ks.gov

**Keywords:** sustainability, community, chronic disease prevention, lifestyle change programs, mixed methods

## Abstract

Community-based chronic disease prevention programs can have long-term, broad public health benefits. Yet, only 40 to 60% of evidence-based health programs are sustained. Using established frameworks and evidence-based tools to characterize sustainability allows programs to develop structures and processes to leverage resources effectively to sustain effective program activities and systems. This study used a mixed-methods, partner-engaged approach to identify barriers and facilitators to sustaining a community network (the Alliance program) aimed to increase participation in evidence-based lifestyle change programs delivered in the community. Surveys and qualitative interviews were conducted with the Alliance partners based on the Program Sustainability Assessment Tool and Consolidated Framework for Implementation Research. Overall, partners felt Alliance had a high capacity for sustainability. Strategic planning, communication, and partnerships were areas partners prioritized to improve the potential for sustaining the program. Results informed the co-development of a sustainability action plan. This paper furthers our understanding of factors critical for the sustainability of community-based programs for chronic disease prevention and health equity and presents a process for developing action plans to build sustainability capacity.

## 1. Introduction

Evidence-based chronic disease prevention programs that are offered in the community can have long-term, broad public health benefits and reduce health disparities [1]. Despite substantial investments in the development and implementation of these programs, sustainability can be challenging due to shifts in priorities, fiscal changes, or political climates [2]. Research shows that only 40–60% of health promotion programs are sustained [3,4]. When initial implementation supports or resources are withdrawn, program delivery is diminished or discontinued entirely. Therefore, long-term positive health impacts are often not realized from public health programs or are not achieved equitably across a range of settings and populations, which may further health disparities. This may be especially true for programs targeting chronic diseases, such as diabetes and heart disease, that often require more intensive, maintained programming for mitigation [5]. Furthermore, the frequent discontinuation of effective programs may result in distrust and diminished community support that are critical to engaging community members, especially those in historically marginalized populations, in effective public health programs [6].

Research on ways to improve public health program sustainability has grown over the past decade [7]. There have been substantial increases in knowledge, frameworks, and tools to promote longer-term programs [2,4]. Program sustainability capacity is defined as “the ability to maintain programming and its benefits over time” [2]. Using established frameworks and evidence-based tools to characterize sustainability allows programs to develop structures and processes to leverage resources effectively to sustain effective program activities and systems [8,9]. Sustainability can help to ensure long-term impact, enhance accountability, promote adaptive management, and support learning and improvement. Ultimately, this practice ensures that interventions and programs are designed and implemented with a focus on these core areas and ensures resources needed for investment in these interventions. The Diabetes Prevention Program (DPP) established in 2010 is an example of a sustained program; yet, its reach and impact on under-resourced residents and health disparities are less clear [10].

The St. Louis region is the largest metropolitan area in Missouri and, like many urban areas, faces significant racial and economic disparity challenges. Poverty affects close to 1 in 3 African Americans versus less than 1 in 10 whites in St. Louis City and St. Louis County [11]. The St. Louis Promise Zone and other impoverished areas in the region suffer from inadequate neighborhood and food environments and lack of access to healthcare. Such environments make people in these areas at even higher risk for chronic disease and less likely to attend evidence-based programs to help prevent their disease [12]. Despite seven organizations offering evidence-based lifestyle change programs (LCPs) at multiple locations in St. Louis and virtually, there is a need to create and sustain better referral, enrollment, and retention to support participation among those living in impoverished, high-risk areas.

The Alliance program, which began in 2018, is a collaboration among healthcare, public health, and community organizations [13]. The Alliance was formed to improve referral, enrollment, and successful completion of evidence-based LCPs, particularly among under-resourced residents, to reduce health disparities in St. Louis. The program focuses on two evidence-based LCPs—the DPP and the Blood Pressure Self-Monitoring program. The DPP is an evidenced-based LCP that has demonstrated effective lifestyle change to prevent or delay the onset of type 2 diabetes. The Blood Pressure Self-Monitoring program was developed by the Centers for Disease Prevention and Control (CDC) to help participants measure their blood pressure correctly and consistently and educate them on healthy eating [14]. The self-monitoring of blood pressure is supported by numerous national agencies (e.g., American Heart Association) and can improve the management of hypertension. The Alliance provides a training platform for frontline workers (e.g., community health workers) to help them engage with patients, refer them to evidence-based LCPs, and assess social barriers that might keep them from attending or successfully completing LCPs. The Alliance also partners with community agencies who could provide resources for social barriers such as food, transportation, childcare, or broadband internet for program participants.

Sustainability of the collaborations and systems of the Alliance will help extend its positive impact. In planning for implementation beyond the initial 5-year project funded by the CDC [15], it was critical to systematically assess factors impacting the program’s sustainability capacity and develop a sustainability plan with actionable strategies. The purpose of this article is to present results of a mixed-methods, partner-engaged approach to identify barriers and facilitators to sustainability and develop a sustainability plan for the Alliance program.

## 2. Materials and Methods

This project used a parallel convergent mixed-methods approach that simultaneously collected quantitative survey data and qualitative interview data to provide a holistic understanding of sustainability of the Alliance program [16]. This understanding was used to co-develop a sustainability plan. This study was approved by the Institutional Review Board at Washington University in St. Louis, IRB # 202207142.

### 2.1. Quantitative Data Collection and Analysis

A survey was distributed electronically via REDCap to all Alliance partners (Table 1) including community health workers, clinicians, community resource coordinators (termed “frontline workers” for this paper), organization managers, program leadership, and funders (N = 74). After the original invitation email, we sent two subsequent reminders in weeks two and three. Participants were offered a USD 20 gift card to a local grocery store as an incentive. There were two main components to the survey: (1) assessment of sustainability capacity based on the 8 domains in the Program Sustainability Framework [2]; and (2) assessment of the inner-organizational factors using validated survey items from the Consolidated Framework for Implementation Research (CFIR) [17].

The Program Sustainability Framework identifies a set of organizational and contextual domains that help build the capacity for maintaining a program. Sustainability capacity is defined as the ability to maintain programming and its benefits over time. To improve this capacity, it is important to strengthen structures and processes that exist within the program. The first step is to build an understanding of the factors that impact the sustainability capacity of a program using the eight key domains: environmental support, funding stability, partnerships, organizational capacity, program evaluation, program adaptation, communication, and strategic planning [2]. The Program Sustainability Assessment Tool (PSAT) was used to collect quantitative data across the eight domains [2]. The PSAT has established reliability for public health programs and has been applied to a variety of public health programs, such as tobacco control, community health, and healthcare programs [18,19,20]. PSAT questions used a 7-point Likert Scale to assess the extent to which each respondent and organization agreed with a series of statements. Scores of 7 indicate agreeing “to a very great extent”, while scores of 1 indicate agreeing “little to no extent”. Responses of 8, indicating “I don’t know/unsure”, were coded as missing and removed prior to quantitative analysis. 

The CFIR is a conceptual framework that was developed to guide the systematic assessment of multi-level contextual factors that may influence the implementation and effectiveness of an intervention or program [21,22]. CFIR assesses barriers and facilitators at five levels: intervention characteristics, outer setting, inner setting, characteristics of individuals, and process of implementation. This study uses a CFIR-derived, validated, and reliable survey, developed by Fernandez and colleagues (2018), to measure inner-organizational factors that may impact implementation and sustainability [17]. While the PSAT includes organizational capacity, we used this CFIR-derived measure to further understand three inner-setting factors (implementation climate, culture stress, and available resources) that may be particularly relevant to the sustainability of the Alliance. CFIR questions were asked on a 5-point Likert Scale (1 = strongly disagree to 5 = strongly agree) to assess the extent of agreement with a series of statements about the respondent’s organizational climate, culture stress, and resources. Higher scores indicate a more positive implementation environment, with culture stress reverse-coded.

Survey results were analyzed using Microsoft Excel using published methods [2]. We calculated means for each item. Domain means were generated by averaging item scores for each of the PSAT and CFIR domains. Items within each domain were bolded if they scored below the domain mean to indicate potential areas for improvement. An overall sustainability score was generated by averaging the 8 PSAT domain scores. Scores were also calculated for each organization by averaging item scores for each respondent within an organization and averaging within each domain. Standard deviations were calculated to show variability within and across organizations.

### 2.2. Qualitative Data Collection and Analysis

We developed interview questions to expand on the PSAT’s eight critical sustainability domains to assess the perception of maintaining the networks and systems developed through the Alliance program beyond the funded grant period. The evaluation team created a general interview guide with a core set of questions, and then added questions tailored for the roles of frontline workers and managers/leaders. We pilot-tested the interview questions internally and with one Alliance partner. No substantive changes to the interview guides were made.

The research team conducted interviews via Zoom video conference. Participants were offered a USD 20 gift card to a local grocery store for participating. The conversations were audio-recorded and transcribed verbatim. After an initial reading of all transcripts, two team members developed a coding tool which was then tested on two transcripts and finalized. Two team members independently coded each transcript and met to ensure inter-rater reliability and address reflexivity in their qualitative interpretation. In these meetings, inconsistencies were reviewed, discussed, and rectified. Coded text was summarized into themes based on PSAT domains. Interview results were presented in our Alliance monthly Zoom meeting with all partners to ensure our analysis and summary accurately captured their perspectives.

### 2.3. Partner-Engaged Sustainability Action Planning

To generate a sustainability action plan, we conducted a modified version of an evidence-based sustainability action planning process shown to improve sustainability capacity in the eight PSAT domains [23]. To prepare the team, quantitative and qualitative results were presented to Alliance partners during a team meeting and a summary was shared to all partners. Following this presentation, all partners were invited to attend a 3 h interactive in-person session to write our sustainability plan. This session focused on deciding what to sustain, choosing which domains were most important and feasible to address, and writing objectives and clear steps. The group also discussed how the plan would be implemented, monitored, and reassessed.

## 3. Results

A total of 17 (9 frontline workers and 8 managers) of the 74 Alliance partners (23%) participated in a survey about the sustainability of the program. Respondents represented seven (78%) of the partner agencies of Alliance. We asked survey respondents (n = 17) if they would be willing to participate in a one-on-one interview with a member of the Alliance evaluation team. A total of 11 expressed interest and agreed to participate in an interview, with 9 (4 frontline workers and 5 managers) completing the interview process within our timeframe. The interviews lasted an average of 21 min. Quantitative and qualitative PSAT results are presented below by domain in descending order of the average score and in Table 2. The communication domain does not have quantitative results, due to an error in REDCap during data collection that generated unusable data. CFIR survey results are summarized below and presented in Table 3.

### 3.1. Program Sustainability Assessment Tool (PSAT) Results

**Figure 1:**
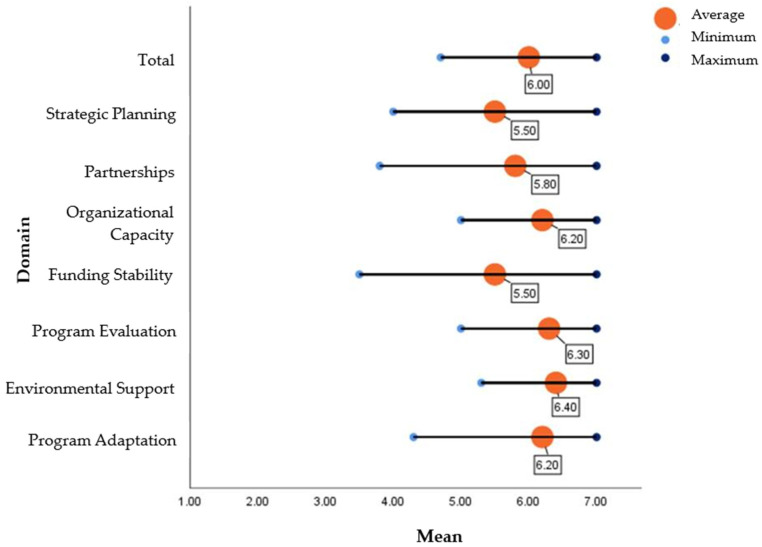
Average sustainability scores by domain.

The PSAT survey results indicated an overall mean score of 6.0 (range: 4.7 to 7.0) for capacity for sustainability based on the 7-point Likert Scale (Table 2, Figure 1). Both managers and frontline staff seemed positive about the potential for sustaining existing work with the Alliance. They noted the long journey of establishing the existing systems of organizational collaboration and support to connect patients into evidence-based disease prevention and management programs. The Alliance frontline staff most often cited the benefits to the community as the reason it should continue. They noted the importance of providing ways to improve health and well-being in places where health inequities exist, and community stakeholder investment.

#### 3.1.1. Environmental Support

Environmental support was the highest ranked domain (mean = 6.4). The item “The Alliance has strong champions with the ability to garner resources” fell slightly below the domain mean (6.2). Having a supportive internal and external climate for the Alliance work is vital to sustainability. One related issue that emerged qualitatively in both frontline staff and managers was the impact of the COVID-19 pandemic. The Alliance brought organizations together, but the COVID pandemic weakened the collaborations and caused a setback in progress. Despite the setbacks, one manager noted the patience that partners had throughout the process of keeping Alliance work going during this time.

#### 3.1.2. Program Evaluation

The mean score for program evaluation was 6.3, with no items scoring below the mean. The importance of using data for the Alliance and its sustainability was noted yet was also perceived as under-appreciated for its contribution by partners. The assessment of social determinants of health was acknowledged by managers as a challenge for frontline workers but necessary to demonstrate the success of the Alliance. One manager mentioned that quarterly meeting updates should include more qualitative data or success stories to get partners engaged in evaluation.

#### 3.1.3. Organizational Capacity

Internal support and resources are needed to effectively manage and sustain programs. This domain scored relatively high with a mean score of 6.2. The item that scored below the mean was the “organization has adequate staff to complete the Alliance goals” (5.8). Qualitatively, it was noted that to achieve Alliance goals and utilize the consistent systems and practices of the Alliance, additional frontline staff are needed. Even when managers were positive about the sustainability of Alliance, they mentioned the silos in which organizations work. Without prioritization and buy-in within these organizations, Alliance activities might be difficult to maintain. Less tangible resources such as capacity for and enthusiasm for the Alliance were mentioned. Regrouping after COVID took time and some momentum was lost. Re-establishing the energy that existed early on may help Alliance in the future. Relatedly, the lack of consistent systems (e.g., electronic health records or social service resource locators) is a significant barrier to collective Alliance work. This was not only noted about referrals into programs, but also for the differences in assessing and addressing social determinants of health which is core to Alliance work.

#### 3.1.4. Program Adaptation

Program adaptation scored high with a mean of 6.2. Items that scored below the mean were “the Alliance adapted to new science” (6.1) and “the Alliance makes decisions about which components are ineffective and should not continue” (6.0). The interview participants talked about the importance of Alliance adaptability and flexibility. All of those interviewed mentioned how the COVID-19 pandemic resulted in adaptations to the Alliance program. Managers mentioned that the move to virtual programs facilitated an acceptance of telehealth, which was a strong benefit to the communities and Alliance as a whole. However, they noted that the pandemic hampered frontline workers’ ability to refer patients due to competing priorities and reduced interactions with the community.

Partners also suggested that Alliance could adapt to better connect with other organizations that provide social services in future efforts. They also mentioned immense needs in the communities they serve, some of which Alliance tries to address and others which were outside of the scope of the project. A broader need for both transportation and food support was noted by several staff and noted as essential for success in disease prevention and management programs. Several frontline staff also talked about mental health needs, especially in the post-COVID era. Several mentioned the need for addressing basic social needs in addition to other Alliance work, especially since many of the people in the program’s target population lack basic tools that would help them be more successful in an LCP.

#### 3.1.5. Partnerships

Partnerships had a mean score of 5.8. Two items scored below the mean: “the Alliance communicates with community leaders” (5.5) and “community leaders are involved with the Alliance” (5.5). This was supported qualitatively as partners felt the Alliance has many internal and external partners, and these partnerships were described as a benefit to the program by all participants. Managers who were interviewed perceived the development and continuation of collaborations and partnerships as crucial to current and future Alliance efforts. The partnerships among public health, healthcare centers, pharmacies, and community resource providers are key to community health impact. They saw Alliance partnerships as key to making a collective impact in their communities, while acknowledging the differences in the ways each organization does this. Frontline staff also talked about the strength in efforts when working collectively and appreciated that Alliance connected them together. There was a common recommendation for the future of Alliance to broaden partnerships within the community. Other community organizations that provide resources, especially food resources, would be helpful in broadening the scope and reach of Alliance.

#### 3.1.6. Strategic Planning

Strategic planning scored 5.5. Items that were indicated as areas for improvement were “the Alliance has a long-term financial plan” (5.3) and “the Alliance’s goals are understood by all partners” (5.0). Qualitative interviews highlighted the need for internal communication that may impact program effectiveness and help build and maintain buy-in with stakeholders. Both managers and frontline workers perceived the need for better ways for the group to communicate within Alliance. More in-person meetings (versus videoconference) were suggested.

#### 3.1.7. Funding Stability

Funding stability (5.5) was among the domains that ranked lowest. Qualitatively, partners expressed that a core component of sustainability is establishing a consistent and reliable financial base for programming. Both frontline staff and managers noted concerns about the continued efforts of Alliance without dedicated grant funds. They noted fears that regardless of how good the work is, it may be difficult to keep the group together without the grant funding. Several participants mentioned that some frontline worker positions are funded by the project, so securing these jobs is integral to sustained Alliance success.

#### 3.1.8. Communication

Participants presented an understanding that external communications build greater visibility and support from stakeholders in the community. Frontline staff are well integrated into the communities in which they serve and mentioned many ways they reach out to find people who could be referred into the Alliance disease prevention and management programs. They noted the need for more outward-facing communication to help in informing communities about the great work that Alliance does, as well as to recruit other organizations that can help meet the needs of our communities to join the Alliance.

### 3.2. Consolidated Framework for Implementation Research (CFIR) Inner-Setting Results 

The Implementation Climate, which is the shared receptivity and extent to which the program will be rewarded, supported, and expected within an organization, scored highest with a mean of 4.3 (Table 3). Two items that scored below the mean were “organization staff get the support they need to improve enrollment and retention in behavior support programs and primary medical care” (4.1) and “Increasing enrollment and retention in behavior support programs and primary medical care is a top priority of the organization” (4.2; Table 3). Available resources scored 4.1, with budget or financial resources (3.8) and patient awareness/need (4.0) as identified areas that are lacking. Culture stress—which was reverse-coded—scored lowest with a mean of 3.8. The lowest item mean in this domain was 3.6 for “staff members often show signs of stress and strain”, followed by “the heavy workload here reduces program effectiveness”, (3.8) and “staff frustration is common here” (3.8; Table 3).

**Table 3:** Inner-organizational factors using the Consolidated Framework for Implementation Research.

Domain (Definition)	Domain Mean (SD)	Items	Item Mean (SD)
**Implementation climate** (Shared receptivity and the extent to which the program will be rewarded, supported, and expected within their organization.)	4.3 (0.5)	Our organization staff are expected to help the Alliance meet its goal (improve enrollment and retention in behavior support programs and/or primary medical care)	4.4 (0.5)
		**Organization staff get the support they need to improve enrollment and retention in behavior support programs and primary medical care**	**4.1 (1.0)**
		Organization staff gets recognition for participating in the Alliance.	4.4 (0.6)
		**Increasing enrollment and retention in behavior support programs and primary medical care is a top priority of the organization.**	**4.2 (0.8)**
**Available resources** (Resources dedicated for implementing the program and ongoing operations-e.g., money, training, space, time)	4.1 (0.3)	In general, when there is agreement that change needs to happen we have the necessary support in terms of:	
		**budget or financial resources**	**3.8 (0.7)**
		training	4.3 (0.7)
		staffing	4.1 (0.7)
		The following are available to make Alliance work in our organization:	
		equipment and materials	4.2 (0.6)
		**patient awareness/need**	**4.0 (0.8)**
		frontline buy-in	4.1 (0.9)
**Culture stress** (Perceived stain, stress and role overload)	3.8 (0.2)	I am under too many pressures to do my job effectively	4.1 (0.9)
		**Staff members often show signs of stress and strain**	**3.6 (1.0)**
		The heavy workload here reduces program effectiveness	3.8 (1.1)
		Staff frustration is common here	3.8 (1.0)

All items are on a 5-point Likert Scale (strongly disagree to strongly agree). Higher scores indicate a more positive implementation environment, with culture stress reverse-coded. Bolded items indicate those that scored below the domain mean, indicating areas for developing capacity.

### 3.3. Sustainability Plan Results

Twelve partners attended the action planning meeting and collectively prioritized PSAT domains by importance and feasibility. Funding stability ranked highest in importance, followed by partnerships and communication. Strategic planning, program adaptation, and program evaluation were rated highest for feasibility. The Alliance partners modified the mission, vision, and values of the Alliance to make it clear what was being sustained and generated objectives and activities for the next year (Supplementary Materials). Partnerships, communication, and strategic planning were selected as the three areas of focus for the next year. The team identified what success would look like for each activity (i.e., how it would be monitored), who would be responsible for completing the activity, and when it would be completed. The group also discussed the ongoing nature of this document and how the team may build on this beyond the one-year period.

## 4. Discussion

As part of a broader evaluation of the Alliance program, we used quantitative surveys and qualitative interviews to evaluate sustainability capacity. These data informed the development of a partner-driven sustainability action plan that will increase the likelihood that the Alliance program continues to make an impact on health disparities in St. Louis. Over the project period, the Alliance program engaged with close to 10,000 St. Louis residents making 1715 referrals to LCPs. About half (52%) of those individuals referred lived in the Promise Zone, a low-resourced, impoverished area. Of those referred, 796 were eligible and enrolled in an LCP. Overall, partners felt that the program has a high capacity for sustaining this network of referral and support into LCPs. Maintaining and expanding partnerships, strategic planning, and communication were areas where partners chose to focus on to enhance program sustainability. Activities that expand partnerships may address inner-organization weakness (Table 3) by increasing resource availability and staff. Communication activities are intended to increase awareness and buy-in among community leaders and patients who may benefit from LCPs. Despite some inner-organizational weaknesses, partners felt that there are strong champions and environmental support, evaluation, and organizational leadership capacity for the Alliance program.

The foundation of the Alliance program is partnerships across healthcare, public health, and community organizations. Partners noted the strength of the collective and multidisciplinary work of the Alliance, which is necessary to generate an impact on chronic disease prevention among the most vulnerable populations to reduce health disparities [24,25]. Yet, the expansion of partnerships was included within the sustainability action plan to improve their capacity to adapt to and meet the unique needs of the community (e.g., include organizations offering mental health resources). Partners identified a need for external communication of our programming to further partnerships and awareness among the community. Managers and frontline staff felt they needed more community resources to adequately and equitably address the needs of community members to reduce health disparities. Furthermore, the level of involvement of community leaders and community members was noted as a weakness of the Alliance. Specifically, involving the community in co-developing the program’s goals and sustainability plans from the start was lacking, yet may generate greater buy-in that partners felt was needed. Our partners noted that concerted efforts to build relationships, infrastructure, and systems that fit within the structures and workflows of all partners is necessary to achieve our program’s goals. While there is an abundance of support for partnerships and rhetoric on their advantages in the literature, there is a lack of an evidence base for what makes a good partnership [26]. Future evaluation of the Alliance may consider iterative evaluation of the strength of the Alliance partnership to iteratively monitor and improve this domain.

As indicated in the Program Sustainability Framework, a variety of funding sources improves sustainability [2]. The Alliance Program was funded by a single five-year grant, which was a substantial concern of the partners. While partners felt hopeful that the Alliance has strong champions with the ability to garner resources, they noted a lack of a long-term financial plan. While specific plans for addressing funding stability were not included in the action plan, the team continues to discuss and apply for various opportunities for funding. Communication to external community leaders and the public was prioritized by partners and may support fund-raising or lead to other supports to increase financial stability. The lack of financial support may be most critical for sustaining the frontline workers within each organization and allocating their time to the goals of the Alliance, two areas identified as lacking within the inner-organizational context. Implementation climate describes inner-organizational factors, such as supporting and prioritizing efforts to engage in and improve lifestyle change program efforts, which have been previously found to be related to local public health efforts to engage in equity-oriented chronic disease prevention [27]. The sustainability scores in the current study indicate that this may be another area for participating organizations to examine ways that internal systems and practices could reduce financial costs of the program and further bolster sustainability.

Strategic planning includes internal communication as well as using processes that guide your program’s directions, goals, and strategies [2]. While partners felt that the evaluation results informed program planning and implementation, they also noted that the program may benefit from more data-driven, strategic decisions about which program components to continue, not continue, or adapt. The Alliance used an iterative evaluation approach. It was noted that the monthly partner reports and sharing of successes and barriers during Alliance partner meetings were critical, in conjunction with the quantitative quarterly evaluation data (e.g., number of referrals made to lifestyle change programs). Partners felt that more opportunities for using qualitative data in the evaluation would improve the ability of the program to adapt as needed. Yet, using these data to inform decisions about the program may not have been transparent or inclusive of all partners. Being intentional about and using established frameworks (such as FRAME) [28] to track program adaptations are critical to sustaining a program. To build capacity for strategic planning, partners felt that it was critical to clarify the program’s goals and each partner’s roles and to develop committees that would focus on sustaining different aspects of the program.

A major strength of our study was the mixed-methods application of the PSAT to understand and assess the sustainability of a complex community network for chronic disease prevention [17]. This study contributes to the literature as few studies have applied mixed methods to understand sustainability capacity and used results to develop an action plan with partners. One known study used open-ended survey questions to gather qualitative data [17], but did not conduct in-depth interviews which allowed us to ask follow-up questions that increase understanding. This study expands PSAT’s organizational capacity domain, using a CFIR-derived measure that examines three inner-setting factors (implementation climate, culture stress, and available resources) that may be particularly relevant to sustainability. While this study comprehensively assessed sustainability, the response rate (23%) for the quantitative survey was low and, therefore, may not fully reflect the perspectives of all partners in the Alliance. This may have generated results that were not as meaningful for sustainability action planning. However, survey respondents did represent all the main Alliance organizations and included an almost equal number of frontline workers and managers/program leadership. Furthermore, partners had additional opportunities to provide their perspectives during Alliance monthly meetings in which results were presented and during discussion at the action planning session. The action planning sessions allowed partners to reach a consensus regarding which domains and specific areas were most critical to address. While we present data for all eight PSAT domains, quantitative survey results were not presented for the communication domain, which limits our ability to compare this domain to others. However, qualitative insights provided a clear need for improved internal and external communication. This was further supported by results in the partnership domain that indicated a need to communicate with community leaders and the strategic planning domain which indicated that, internally, not all alliance partners understand the goals of the program.

## 5. Conclusions

Understanding what factors are critical for sustaining effective programs and processes for developing action plans to build sustainability capacity is critical to maximizing community health impact and reducing health disparities. Therefore, understanding and planning for sustainability are of interest to researchers, practitioners, and funders [2,5]. Using established frameworks and evidence-based tools to characterize sustainability allows programs to develop structures and processes to leverage resources effectively to sustain effective program activities and systems. Furthermore, this process allows programs to act more efficiently and improve their ability to maintain efforts over the long-term. While the Sustainability Framework highlights critical domains for sustainability, the factors that are necessary and sufficient to ensure sustainability likely differ for intervention types and contexts [3,29]. Using a mixed-methods approach to examine barriers and facilitators of sustaining a complex community network for chronic disease prevention may further our understanding of factors critical for the sustainability of public health programs for chronic disease prevention and health equity.

## Figures and Tables

**Table 1:** Alliance partners.

Partner	Role(s)
Centers for Disease Control and Prevention	Funding agency
Missouri Department of Health and Senior Services	Program leadership
Gateway Region YMCA	Managers, frontline workers, lifestyle coaches
Missouri Primary Care Association	Managers, frontline workers, clinicians
Missouri Pharmacy Association	Managers, pharmacists
Integrated Health Network	Managers, frontline workers
St. Louis City Health Department	Managers, resource provider
St. Louis County Health Department	Manager, training center, frontline workers
Washington University in St. Louis Saint Louis University	Evaluation leadership
Fit and Food Connection	Community Organization/Provider
Operation Food Search	Community Organization/Provider

**Table 2:** Program Sustainability Assessment Tool (PSAT) survey results and qualitative quotes.

Domain (Definition)	Domain Mean (SD)	Items	Item Mean (SD)	Exemplar Quotes
**Environmental support** (Having a supportive internal and external climate for your program)	6.4 (0.7)	Champions exist who strongly support the Alliance.	6.4 (1.4)	“COVID obviously turned everything upside down. But I think the Alliance did a really good job of pulling out what we could best support for the community in the pandemic and then building out or stepping up”. “I can think of quite a few individuals and organizations that either are directly or have the potential to be [champions for the Alliance]”.
		**The Alliance has strong champions with the ability to garner resources.**	**6.2 (0.9)**	
		The Alliance has leadership support from within your organization	6.7 (0.8)	
		The Alliance has leadership support from outside your organization.	6.4 (1.1)	
**Program evaluation** (Assessing your program to inform planning and document results)	6.3 (0.7)	The Alliance has the capacity for quality program evaluation.	6.3 (1.0)	“I think it’s really difficult to define data, especially as it changes. And each partner may have a different need for looking at different data. So I don’t know if the numbers, or at least the quantitative data, is as helpful. I do think seeing the monthly reports and the updates from other partners is helpful, but more that qualitative data is probably better, or even hearing what successes or barriers others are having is definitely more helpful than the numbers”.
		The Alliance reports short term and intermediate outcomes.	6.4 (0.8)	
		Evaluation results inform program planning and implementation.	6.3 (0.7)	
		Alliance evaluation results are used to demonstrate success to fundings and other key stakeholders.	6.5 (0.7)	
**Organizational capacity** (Having the internal support and resources needed to effectively manage your program)	6.2 (0.6)	The Alliance work is well integrated into the operations of your organization.	6.4 (0.8)	“So I think just really reestablishing that buy-in from leadership…it does feel like perhaps there is a little bit of, I don’t know if it’s a lack of understanding or lack of prioritization or what it is, lack of buy-in, that feels that way now”.
		Organizational systems are in place to support the various Alliance needs.	6.2 (1.3)	
		Alliance leadership (e.g., DHSS, county) effectively articulates the vision of the program to partners.	6.4 (0.8)	
		Leadership efficiently coordinates staff and other resources.	6.4 (0.7)	
		**Your organization has adequate staff to complete the Alliance goals**	**5.8 (1.4)**	
**Program adaptation** (Taking actions that adapt your program to ensure ongoing effectiveness)	6.2 (0.8)	The Alliance adapts strategies as needed.	6.3 (1.1)	“I definitely think that we should tap into the mental health part of our community. I think that there is a direct correlation between your mental health and your physical health and mental health and chronic disease. And I’m not sure exactly what is in place for individuals who come into the program and they express that they want assistance.
		**The Alliance adapts to new science.**	**6.1 (1.1)**	
		The Alliance proactively adapts to changes in the environment.	6.5 (0.7)	
		**The Alliance makes decisions about which components are ineffective and should not continue.**	**6.0 (1.3)**	
**Partnerships** (Cultivating connections between your program and its stakeholders)	5.8 (1.0)	Diverse community organizations are invested in the success of the Alliance.	6.5 (0.9)	“I do think that just the cross collaboration and just being stronger together as a collective is always better than being siloed. You cover more ground, you do better work. And so definitely this is not a who can do what more? It’s never like that. So we’re always motivated to work with others”. “And so I think that can be more, again, opportunities for us to share with the community, whether it’s through board meetings or town hall meetings or through church announcements across the area, whatever. Just getting the word out so that those who have the means and those who share the same passion can also join us”.
		**The Alliance communicates with community leaders.**	**5.6 (1.5)**	
		**Community leaders are involved with the Alliance.**	**5.5 (1.5)**	
		Community organizations are passionately committed to the program.	5.9 (1.2)	
		**The community is engaged in the development of the Alliance goals.**	**5.3 (1.6)**	
**Strategic planning** (Using processes that guide your program’s directions, goals, and strategies)	5.5 (1.1)	The Alliance plans for future resource needs.	6.1 (0.9)	“When you have a group together that has a focused goal, which we all do, we want to help our patients, it strengthens this area, and our patients benefit from it”. “For sustainability past the grant, it’s even facilitating discussions, more discussions among the partners involved”.
		**The Alliance has a long-term financial plan.**	**5.3 (1.4)**	
		The Alliance has a sustainability plan.	5.6 (1.2)	
		**The Alliance’s goals are understood by all stakeholders.**	**5.0 (1.6)**	
		The Alliance clearly outlines roles and responsibilities for all stakeholders.	5.7 (1.2)	
**Funding stability** (Establishing a consistent financial base for your program)	5.5 (1.2)	The Alliance exists in a supportive state economic climate.	5.9 (1.3)	“Our position was created from the grant, so we will actively be looking to securing other funding to continue the position, in order to continue being an Alliance partner or working with Alliance members. But yeah, I feel like we have the capacity, we have the people, it’s just about sustaining us, I guess”. “I’m very hopeful that we can continue to work, but also I’m not naïve. I understand that sometimes it comes down to funding”. “I believe that if there is a way for the grant writers or admin to find funding, the effort is always there to try to keep it going and to properly support people or get donations or whatever. So I can say that, but how many ways there is to bring those resources in and funds in, I’m not sure”
		**The Alliance is funded through a variety of sources.**	**5.4 (1.5)**	
		The Alliance has a combination of stable and flexible funding.	5.6 (1.3)	
		The Alliance has sustained funding.	5.6 (1.2)	
**Communications** (Strategic communication with partners and the public about the program)	(see note)	(see note)	(see note)	“I think for sustainability past the grant, it’s even facilitating discussions, more discussions among the partners involved”. “It doesn’t feel like there are many communication tools. What I can think of is probably Basecamp, which we started using the beginning for training purposes. However, when people use it now, it’s kind of like, what’s the date of this training or what’s the link for this training? So it’s not much cross collaboration or question asking. So maybe there should be something else to help with that, especially for the frontline staff, I feel, since we are trying to problem-solve or trying to make referrals”
**Overall Capacity**	6.0 (0.7)	NA	NA	NA

All items use a 7-point Likert Scale. Bolded items indicate those that scored below the domain mean, indicating areas for developing capacity. Note: an error in the RedCap survey resulted in no data for the PSAT communication domain. We did collect qualitative information on this topic.

## Data Availability

The data presented in this study are available on request from the corresponding author. The data are not publicly available, due to privacy concerns for participating Alliance partners.

## Supplementary Materials

The following supporting information can be downloaded at: https://www.mdpi.com/article/10.3390/ijerph21040463/s1, The St. Louis Alliance Sustainability Plan.
