# Supplementary material for: Sustainability Planning for a Community Network to Increase Participation in Evidence-Based Lifestyle Change Programs: A Mixed-Methods Approach
Source: Int J Environ Res Public Health. 2024 Apr 10;21(4):463. doi: 10.3390/ijerph21040463 (PMC11050027; doi:10.3390/ijerph21040463)
Supplement: Supplementary file 1 [file ijerph-21-00463-s001.zip › ijerph-2920728-supplementary.pdf]

St. Louis Alliance Sustainability Plan: Cultivating a Sense of Momentum and Progress

Background: The Alliance, which began in Missouri in 2019, is a collaboration among healthcare, public health, and community organizations formed to improve referral, enrollment, and successful completion of evidence-based lifestyle change programs (LCPs), particularly among marginalized individuals. The Alliance focuses on addressing social barriers that prevent individuals from accessing appropriate care and living healthy lives as well as a training center for frontline staff to expand their knowledge and cultural humility. The Alliance program and partnerships are funded by the Center for Disease Control and Prevention's Division of Diabetes Translation and Heart Disease & Stroke Prevention 1817 projects that launched in Missouri in January 2019 and ends September 2023. The Original program vision statement was "Optimize health equity across the St. Louis area by improving enrollment, retention, and success in behavior support programs and evidence-based Lifestyle Change Programs. The Alliance will continue working to address the influence of trauma and social determinants within the target population to advance prevention and control of diabetes and cardiovascular disease." This document outlines plans to sustain these efforts for a one year period from September 2023-October 2024.

Sustainability Planning Methods: To inform program sustainability, data was collected from Alliance partners via a quantitative survey and qualitative interviews. After qualitative and quantitative sustainability planning data was presented to Alliance partners, an in-person meeting was held to further develop the action plan. Twelve partners attended the meeting and collectively prioritized PSAT domains by importance and feasibility. The Alliance partners also collaborated to modify the mission, vision and values and generate objectives and activities for the next year. The group discussed the ongoing nature of this document and how the team will use it beyond the one-year period.

New Vision, Mission and Values:

Revised Vision Statement*:* The Vision of the Alliance is to increase the reach and impact of lifestyle change programs among St. Louisans who are at higher risk of chronic diseases.

Revised Mission Statement: The mission of Alliance is to increase referrals, enrollment, and successful completion of evidence-based lifestyle change programs by addressing social barriers of St. Louisans who are under-resourced.

Alliance Values:

- Focusing on high-need areas in St. Louis
- Treating the community with respect and engaging in equitable interactions
- Prioritizing trauma-informed approaches
- Building capacity by expanding resource partners
- Being supportive of Alliance members
- Having a sustained commitment to the community

Areas prioritized by partners to build sustainability capacity: Funding stability ranked the highest on importance, followed by partnerships, and communication. Strategic planning, program adaptation, and program evaluation were rated as the highest for feasibility. Based on discussion, strategic planning, communication, and partnerships were selected to generate specific objectives and activities presented in the tables below.

**Strategic Planning:** Using processes that guide your program's directions, goals, and strategies.								
**Sustainability Objective**: Create an Alliance Guide (digital booklet)								
**Steps to achieve objectives:**	**Who will do the work?[1]**	**What does success look like?**	**What non- financial resources are needed for this step? Where will they come from?**	**Due date**				
				**10/23-12/23**	**1/24-3/24**	**4/24-6/24**	**7/24-9/24**	
**1.** Convene partners to update the Alliance's purpose, mission and vision.	Evaluation Team	Meeting planned and implemented.<br>Mission and vision revised.	none	X				
**2.** Define each partner's roles and our formal structure.	St. Louis County Health Department; Evaluation Team	A list of partner's roles and structure is created, approved and added to the Alliance Guide.	none	X				
**3.** Develop subcommittees to implement different aspects of work (e.g., communications, etc.)		Sub-work topics created (proposed: communications and partnerships).<br>Sub-committees created.<br>Leader for each sub-committee determined.		X				
**4.** Develop plan for Alliance meetings.	Frontline meetings quarterly hosted by St. Louis County Health Department; Manager meeting hosted by St. Louis County Health Department	Calendar of meeting dates created.<br>Meeting logistics and leadership set.	Develop the purpose of these meetings to make clear the benefit/reason for attendance;<br>Consider rolling frontline meetings into existing meeting structure through the coalition;					

**5.** Integrate managers and frontline basecamp. Disseminate and maintain basecamp.	St. Louis County Health Department	New platform chosen and being used by Alliance partners.		X			
**6.** Maintain Alliance Guide and Directory.	Missouri Department of Health and Senior Services	Alliance guide up to date and accessible to all partners.		X	X	X	X

[1] For the purposes of publication, this information was kept broad. Within the internal document, specific individuals were named to ensure specificity and accountability.

**Communications:** Strategic communication with stakeholders and the public about your program							
**Sustainability Objective**: Externally communicate our programming (established in Strategic Planning goal) to the St. Louis community.							
**Steps to achieve objectives:**	**Who will do the work?**	**What does success look like?**	**What non- financial resources are needed for this step? Where will they come from?**	**Due date**			
				**10/23-12/23**	**1/24-3/24**	**4/24-6/24**	**7/24-9/24**
**1.** Develop a communication plan to communicate with participants<br>**a.** Select communication materials and methods (e.g., social media, flyers)<br>b. Determine implementation of the communication plan (e.g., frequency of posts)	Communications committee to be formed	Communication plan created and approved.	Committee formed		X		
**2.** Develop an Alliance logo.	St. Louis County Health Department	Logo developed.	Design expertise- external company	X			

**3.** Implement communications plan.	Communications committee to be formed	Communication materials disseminated. # of individuals reached.				X	X

**Partnerships:** Cultivating connections between your program and its stakeholders							
**Sustainability Objective**: Maintain existing partnerships and develop relevant new partners to achieve the Alliance mission.							
**Steps to achieve objectives:**	**Who will do the work?**	**What does success look like?**	**What non- financial resources are needed for this step? Where will they come from?**	**Due date** [In the appropriate quarter, enter a specific date by which the activity must be completed.]			
				**Q1**	**Q2**	**Q3**	**Q4**
1. Identify gaps in our services/activities and identify potential partners to fill gaps.	Partnerships committee to be formed	Gaps identified.<br>A list of potential partners created.	Committee				X
2. Connect new partners to Alliance.	All partners; partnerships committee	Partners engage with Alliance.	Methods for connecting partners.<br>Alliance Guidebook (Objectives 1)				X
3. Disseminate communication products to new partners.	Communications committee	One information sheet about Alliance was shared.	Communication products (Objective 2)				X
4. Add new partners to internal documents and communication platform.	St. Louis County Health Department	Up to date Alliance Guide, directory and communication platform.					X
